# 1,25-Dihydroxyvitamin D Modulates Antibacterial and Inflammatory Response in Human Cigarette Smoke-Exposed Macrophages

**DOI:** 10.1371/journal.pone.0160482

**Published:** 2016-08-11

**Authors:** Nele Heulens, Hannelie Korf, Carolien Mathyssen, Stephanie Everaerts, Elien De Smidt, Christophe Dooms, Jonas Yserbyt, Conny Gysemans, Ghislaine Gayan-Ramirez, Chantal Mathieu, Wim Janssens

**Affiliations:** 1 Laboratory of Respiratory Diseases, Department of Clinical and Experimental Medicine, Katholieke Universiteit Leuven, Leuven, Belgium; 2 Laboratory of Clinical and Experimental Endocrinology, Department of Clinical and Experimental Medicine, Katholieke Universiteit Leuven, Leuven, Belgium; Nihon University School of Medicine, JAPAN

## Abstract

Cigarette smoking is associated with increased inflammation and defective antibacterial responses in the airways. Interestingly, vitamin D has been shown to suppress inflammation and to improve antibacterial defense. However, it is currently unknown whether vitamin D may modulate inflammation and antibacterial defects in human cigarette smoke (CS)-exposed airways. To explore these unresolved issues, alveolar macrophages obtained from non-smoking and smoking subjects as well as human cigarette smoke extract (CSE)-treated THP-1 macrophages were stimulated with 1,25-dihydroxyvitamin D (1,25(OH)_2_D) to address inflammatory and antibacterial responses. Although basal levels of inflammatory cytokines and chemokines did not differ between non-smoking and smoking subjects, 1,25(OH)_2_D did reduce levels of IL-6, TNF-α and MCP-1 in alveolar macrophages in response to LPS/IFN-γ, although not statistically significant for TNF-α and IL-6 in smokers. CSE did not significantly alter vitamin D metabolism (expression levels of CYP24A1 or CYP27B1) in THP-1 macrophages. Furthermore, stimulation with 1,25(OH)_2_D reduced mRNA expression levels and/or protein levels of IL-8, TNF-α and MCP-1 in CSE-treated THP-1 macrophages. 1,25(OH)_2_D did not improve defects in phagocytosis of *E*. *coli* bacteria or the oxidative burst response in CSE-treated THP-1 macrophages or alveolar macrophages from smokers. However, 1,25(OH)_2_D significantly enhanced mRNA expression and/or protein levels of the antimicrobial peptide cathelicidin in alveolar macrophages and THP-1 macrophages, independently of CS exposure. In conclusion, our results provide the first evidence that vitamin D could be a new strategy for attenuating airway inflammation and improving antibacterial defense in CS-exposed airways.

## Introduction

In recent years, it has become apparent that the active form of vitamin D, 1,25-dihydroxyvitamin D (1,25(OH)_2_D), is not only important for calcium and bone homeostasis, but also exerts important innate immunomodulatory functions [1]. 1,25(OH)_2_D has been shown to inhibit the production of inflammatory cytokines and chemokines *in vitro* in response to various inflammatory and/or infectious stimuli [2–5]. In addition to these anti-inflammatory actions, 1,25(OH)_2_D may improve antibacterial defense by stimulating phagocytosis [6–8] as well as by enhancing the production of reactive oxygen species (oxidative burst) [9] and antimicrobial peptides (including cathelicidin) [10,11], which both are important for the efficient killing of bacteria.

Cigarette smoking is a global epidemic and a major risk factor for several life-threatening diseases, including chronic obstructive pulmonary disease (COPD). Alveolar macrophages are crucial in initiating the inflammatory response to cigarette smoke (CS) by releasing inflammatory cytokines and chemokines, such as IL-8 and MCP-1 [12]. This then recruits additional inflammatory cells, including monocytes and neutrophils, to the lungs, which amplifies the inflammatory response. Alveolar macrophages are furthermore important resident phagocytes in the lung, contributing to the clearance of infections [13]. However, CS has been shown to impair antibacterial defense, including the phagocytic uptake of bacteria by macrophages, as demonstrated by *in vitro* as well as *in vivo* animal research [14–19].

Given the anti-inflammatory and antibacterial functions of 1,25(OH)_2_D, it is tempting to speculate that 1,25(OH)_2_D could counteract these effects of CS. However, few studies have suggested that CS could affect vitamin D metabolism by increasing CYP24A1 (24-hydroxylase, catabolizing enzyme that degrades 1,25(OH)_2_D) [20] or decreasing CYP27B1 (1α-hydroxylase, activating enzyme leading to formation of 1,25(OH)_2_D) [21]. As CYP24A1, CYP27B1, but also the vitamin D receptor (VDR) are expressed locally within the lungs [22], such as in alveolar macrophages, these effects of CS on vitamin D metabolism could potentially limit immunomodulatory effects of vitamin D within the respiratory tract. As such, it is still unclear whether 1,25(OH)_2_D modulates pulmonary inflammatory responses and/or antibacterial defects in CS-exposed airways. As macrophages are major players in inflammatory and antibacterial responses in the airways, we investigated the effect of 1,25(OH)_2_D on i) *ex vivo* responses of alveolar macrophages from smoking subjects (compared to non-smoking subjects), and ii) THP-1 macrophages exposed to cigarette smoke extract (CSE).

## Materials and Methods

Experiments involving human samples were approved by the Ethical Committee of University Hospital UZ Leuven (S54148) and all subjects gave informed, written consent.

### Bronchoalveolar lavage (BAL) of non-smoking and smoking subjects

Non-smoking (n = 10) and smoking (n = 11) subjects, undergoing bronchoscopy for diagnostic reasons, were recruited. Non-smokers were defined as subjects with a normal lung function who never smoked or stopped smoking for more than 5 years, while smokers were defined as subjects with a normal lung function who were current smokers at the moment of BAL sampling (34±5 pack-years). Normal lung function was defined as forced expiratory volume in 1 second (FEV1)% > 80% and Tiffeneau-index (FEV1 over forced vital capacity (FVC; FEV1/FVC) > 0.7. Non-smokers and smokers were matched for age (non-smoker: 66.4±3.14 years; smokers: 59.8±2.29 years; p = 0.0982) and gender (non-smoker: 60% male; smoker: 64% male; p = 0.8639). To reduce bias from bacterial colonization and chronic inflammation, COPD patients were a priori excluded. As a consequence, none of the subjects were taking oral or inhaled corticosteroids or showed any signs of acute respiratory infection at the moment of BAL sampling. BAL was performed according to standard procedures [23]. BAL consisted of four aliquots of 50 ml sterile saline instilled in the right middle lobe or lingula. BAL samples were filtered and cells were resuspended in culture medium (RPMI 1640 medium-Glutamax supplemented with 10% fetal calf serum (FCS), 100 U/ml penicillin, 100 μg/ml streptomycin, and 2.5 mg/ml fungizone). Alveolar macrophages were isolated by incubating BAL cells for 2 hours at 37°C to allow adherence of macrophages. Afterwards, non-adherent cells were removed by repeatedly replacing the medium. Alveolar macrophages were stimulated for 48h with 10^-8^M 1,25(OH)_2_D (Sigma-Aldrich, St-Louis, MO, USA). The dose and exposure time of 1,25(OH)2D were based upon literature [24,25]. In certain conditions, alveolar macrophages were additionally stimulated for 24h with a combination of LPS (1 μg/ml) and IFN-γ, (100 IU/ml), a strong inflammatory stimulus [25]. To correct for the different number of alveolar macrophages in each sample, all samples were diluted to 2x10^6^ cells/ml with culture medium.

### THP-1 macrophages

As the number of BAL samples obtained from non-smoking and smoking subjects was limited, additional experiments were performed on the THP-1 monocytic cell line, differentiated into macrophages, which represent a useful model to study alveolar macrophage responses [26].

#### Differentiation and stimulation of THP-1 monocytic cell line

The THP-1 human acute monocytic leukemia cell line (ATCC, Manassas, VA, USA) was cultured in RPMI medium supplemented with 10% fetal calf serum (FCS), 100 U/ml penicillin, 100 μg/ml streptomycin, 2 mM L-glutamine and 2.5 μg/ml fungizone at 37°C (5% CO_2_). To induce differentiation into adherent macrophages, THP-1 cells were stimulated overnight with phorbol 12-myristate 13-acetate (PMA; Sigma-Aldrich) (30 ng/ml) and were allowed to rest for two additional days. THP-1 macrophages were stimulated for 16h with CSE, followed by an additional 24h stimulation with 10^-8^M 1,25(OH)_2_D (1,25(OH)_2_D post-treatment). For certain experiments, THP-1 macrophages were also pre-treated with 1,25(OH)_2_D (Methods in S1 File).

#### Preparation of CSE

CSE was generated by bubbling the smoke of 1 cigarette (3R4F research cigarettes, Kentucky Tobacco Research and Development Center, University of Kentucky) through 10 ml of culture medium. The pH was adjusted to 7.4 and CSE was filtered through a 0.2 mm pore filter (Millipore). The resulting solution was considered 100% CSE. CSE was freshly prepared and used within 30 minutes. To ensure standardization between different preparations of CSE, the absorbance was adjusted to OD 0.4 at 340 nm.

#### Cytotoxicity

Cytotoxicity due to CSE exposure was evaluated using the MTT (3-(4,5-dimethylthiazol-2-yl)-2,5-diphenyltetrazolium bromide) assay. Cells were incubated for 3h with MTT dissolved in HBSS (0.5 mg/ml). The precipitated formazan product was then dissolved in DMSO and absorbance was recorded at a 550 nm wavelength and a reference wavelength of 655 nm. Results are expressed as a percentage of the control values (Figures A and B in S1 File).

### Inflammatory response

#### mRNA and protein levels of inflammatory cytokines and chemokines

Protein levels of IL-8, TNF-α, MCP-1 and IL-6 were measured in cell-free supernatants of alveolar macrophage and THP-1 macrophage cultures using the Mesoscale Discovery (MSD) technology, according to the manufacturer’s instructions. mRNA expression levels of IL-8, TNF-α, MCP-1 and IL-6 in THP-1 macrophages were determined with real-time PCR. Therefore, a constant amount of 1 μg of RNA was reverse transcribed with Superscript III reverse transcriptase (Invitrogen, Life Technologies, Lennik, Belgium). The quantitative PCR amplification reaction was performed using a thermal cycler (Eco Real-Time PCR system, Illumina). Primer sequences are shown in Table 1. Glyceraldehyde 3-phosphate dehydrogenase (GAPDH) was used as housekeeping gene and data were normalized using the comparative cycle threshold (Ct) method.

**Table 1:** Primers used for quantitative PCR analysis.

Target	Sequence
GAPDH	• 5’-TGGTATCGTGGAAGGACTCA-3' (FW) • 5’-CCAGTAGAGGCAGGGATGAT-3' (RV)
IL-8	• 5’-CATCTTCACTGATTCTTGGATACC-3’ (FW) • 5’-TGTCTGGACCCCAAGGAA-3’ (RV)
TNF-α	• 5’-TCAGCTTGAGGGTTTGCTAC-3’ (FW) • 5’-TGCACTTTGGAGTGATCGG-3’ (RV)
MCP-1	• 5’-GCCTCTGCACTGAGATCTTC-3’ (FW) • 5’-AGCAGCCACCTTCATTCC-3’ (RV)
IL-6	• 5’-TTCTGTGCCTGCAGCTTC-3’ (FW) • 5’-GCAGATGAGTACAAAAGTCCTGA-3’(RV)
Cathelicidin	• 5’-GGGCTCCTTTGACATCAGTT-3’ (FW) • 5’-AGCAGGGCAAATCTCTTGTT -3’ (RV)
VDR	• 5’-GATTGGAGAAGCTGGACGAG-3’ (FW) • 5’-GTTCGTGTGAATGATGGTGGA-3’ (RV)
CYP27B1	• 5’-CGCACTGTCCCAAAGCTG-3’ (FW) • 5’-CGGAGCTTGGCAGACATC-3’ (RV)
CYP24A1	• 5’-GTGACCATCATCCTCCCAAA-3’ (FW) • 5’-AGTATCTGCCTCGTGTTGTATG-3’ (RV)

FW: forward primer; RV: reverse primer

### Antibacterial response

#### Phagocytosis and oxidative burst

The phagocytic and oxidative burst capacity was assessed by flow cytometry using an adaptation of the commercial kit BURSTTEST (Orpegen Pharma, Heidelberg, Germany). Briefly, THP-1 macrophages or cells from human BAL samples were incubated for 2 hours at 37°C with *pH*-rodoRed-labeled *E*. *coli* bacteria (*pH*rodo^TM^ Red *E*. *coli* Bioparticles^®^ Phagocytosis kit for flow cytometry, Invitrogen), previously opsonized with *E*. *coli* Bioparticles^®^ opsonizing reagent (Invitrogen). Afterwards, the fluorogenic substrate rhodamine was added for an additional 20 minutes at 37°C. Samples were acquired on a Gallios flow cytometer (Beckman Coulter, Brea, CA, USA) and analyzed with the Kaluza software (Beckman Coulter). For human BAL samples, alveolar macrophages were identified on flow cytometry by staining BAL cells with fluorochrome-labeled monoclonal antibodies against CD11b, CD33 and HLA-DR (eBioscience) for 20 minutes at 4°C.

#### mRNA expression levels and protein levels of cathelicidin

mRNA expression levels of cathelicidin in THP-1 macrophages were determined with real-time PCR, as described above. Primer sequences are shown in Table 1. Levels of cathelicidin in cell-free supernatants of alveolar macrophage and THP-1 macrophage cultures were measured with the human LL-37 ELISA kit (Hycult biotech), according to the manufacturer’s instructions.

### mRNA expression levels of proteins in the vitamin D pathway

mRNA expression levels of VDR, CYP27B1 and CYP24A1 in THP-1 macrophages were determined with real-time PCR, as described above. Primer sequences are shown in Table 1.

### Statistical analysis

Data were analyzed using SAS software version 9.3 and are presented as mean±SEM. Differences between groups were analyzed using a two-way ANOVA with a Tukey-Kramer *post-hoc* test for multiple group comparison. A mixed model was applied to account for independent experiments (THP-1 macrophages) or individual patient samples (human alveolar macrophages). Differences were considered significant when p-values were less than 0.05.

## Results

### 1,25(OH)_2_D inhibits *ex vivo* inflammatory responses of alveolar macrophages from both non-smokers and smokers

**Fig 1:**
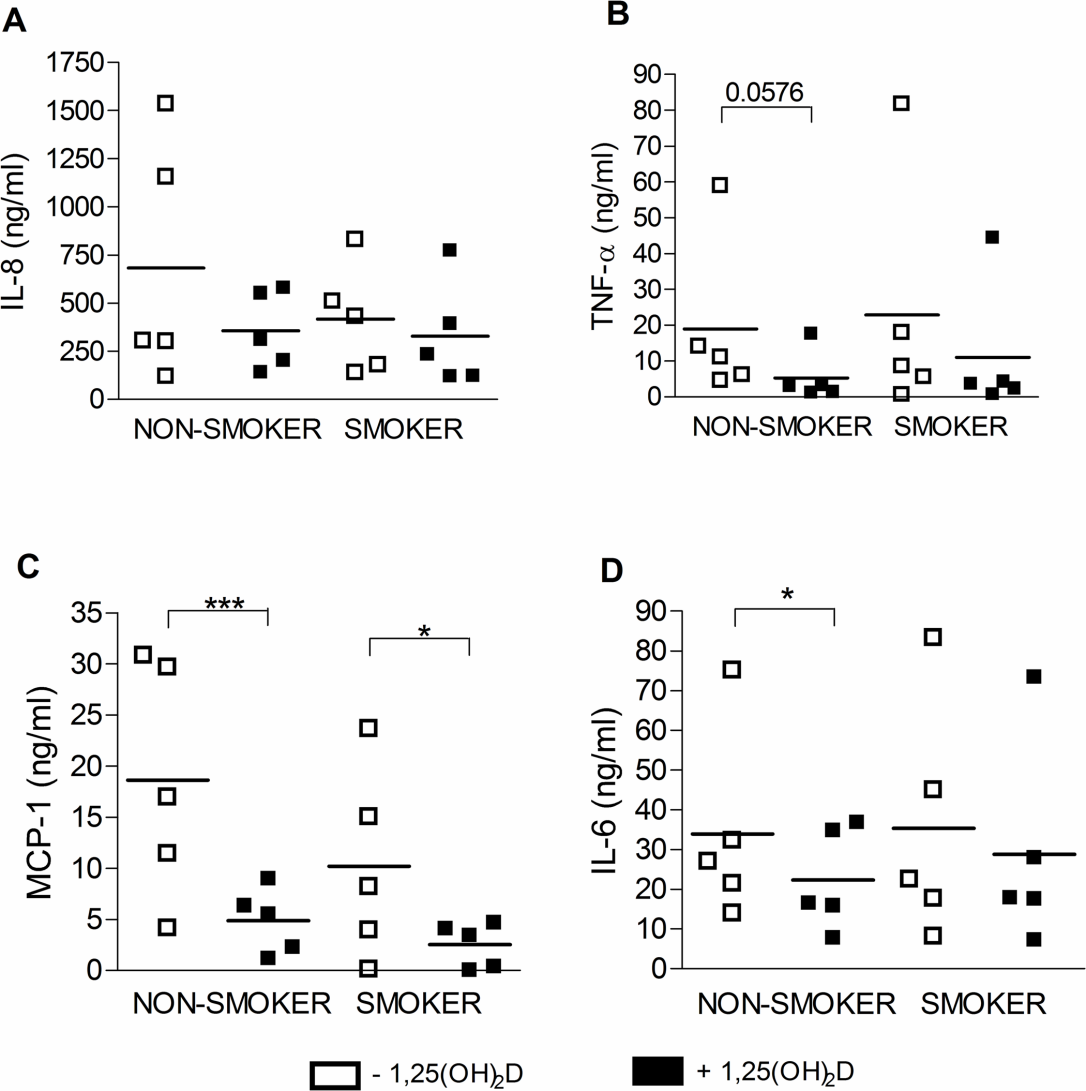
Effect of 1,25(OH)_2_D on the *ex vivo* inflammatory response of alveolar macrophages from non-smoking and smoking subjects. Alveolar macrophages from non-smokers (n = 5) or smokers (n = 5) were stimulated for 48h with 10 nM 1,25(OH)_2_D or vehicle, followed by an additional stimulation for 24h with LPS/IFN-γ. Levels of (A) IL-8, (B) TNF-α, (C) MCP-1 and (D) IL-6 were measured in supernatants of alveolar macrophage cultures. mean±SEM. *p<0.05, ***p<0.001.

We aimed to investigate whether 1,25(OH)_2_D influences *ex vivo* inflammatory and/or antibacterial responses of alveolar macrophages from smoking subjects. Therefore, BAL samples were obtained from age- and gender-matched non-smoking and smoking subjects. No differences were observed for basal levels of IL-8, TNF-α, MCP-1 and IL-6 produced by alveolar macrophages from non-smokers or smokers. Treatment with 1,25(OH)_2_D did not alter these basal levels released by alveolar macrophages from non-smoking or smoking subjects (data not shown). However, when alveolar macrophages were additionally stimulated for 24h with a combination of LPS and IFN-γ, a strong inflammatory stimulus, 1,25(OH)_2_D significantly reduced levels of TNF-α, MCP-1 and IL-6 in alveolar macrophages from non-smoking subjects in response to LPS/IFN-γ (Fig 1). Similar trends were observed for alveolar macrophages from smoking subjects, although not statistically significant for levels of TNF-α and IL-6 (Fig 1). No significant differences were observed for levels of IL-8 released by alveolar macrophages from both non-smokers and smokers (Fig 1).

### 1,25(OH)_2_D does not affect *ex vivo* phagocytic or oxidative burst responses, but enhances release of cathelicidin from alveolar macrophages from both non-smoking and smoking subjects

**Fig 2:**
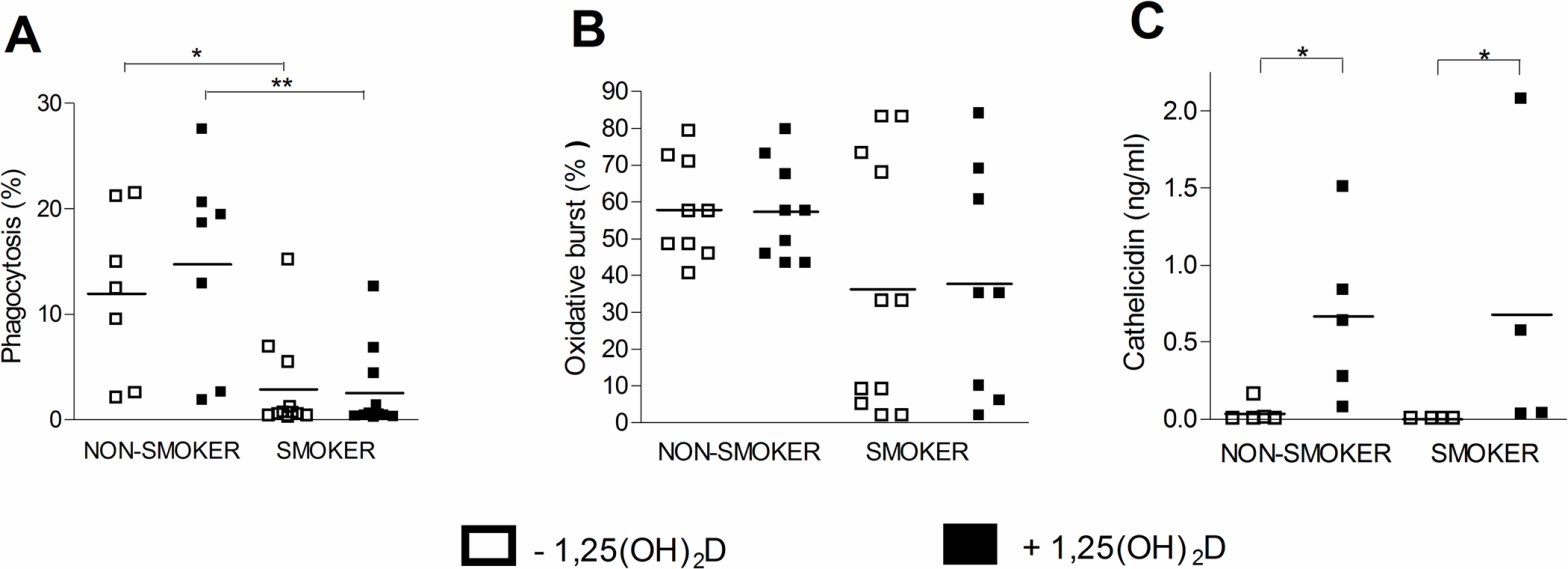
Effect of 1,25(OH)_2_D on *ex vivo* antibacterial response of alveolar macrophages from non-smoking and smoking subjects. Alveolar macrophages from non-smokers or smokers were stimulated for 48h with 10 nM 1,25(OH)_2_D or vehicle. (A) Phagocytosis and (B) oxidative burst by alveolar macrophages from non-smokers (n = 7) and smokers (n = 11) following bacterial challenge. Graphs show the percentage of alveolar macrophages that have internalized *E*. *coli* bacteria (A) or have produced reactive oxygen species (B). (C) Cathelicidin levels were measured in supernatants of alveolar macrophage cultures from non-smokers (n = 5) and smokers (n = 5). mean±SEM. *p<0.05, **p<0.01.

The phagocytic uptake of *E*. *coli* bacteria was significantly decreased in alveolar macrophages from smokers compared to non-smokers (Fig 2A). The production of reactive oxygen species (oxidative burst) by alveolar macrophages was also decreased in smokers compared to non-smokers, although not reaching statistical significance (Fig 2B). Stimulation with 1,25(OH)_2_D did not significantly alter phagocytic or oxidative burst responses of alveolar macrophages from non-smoking and smoking individuals. In contrast, stimulation with 1,25(OH)_2_D significantly enhanced the release of the antimicrobial peptide cathelicidin from alveolar macrophages to the same extent in both non-smokers and smokers (Fig 2C).

### 1,25(OH)_2_D inhibits the inflammatory response in CSE-treated THP-1 macrophages

**Fig 3:**
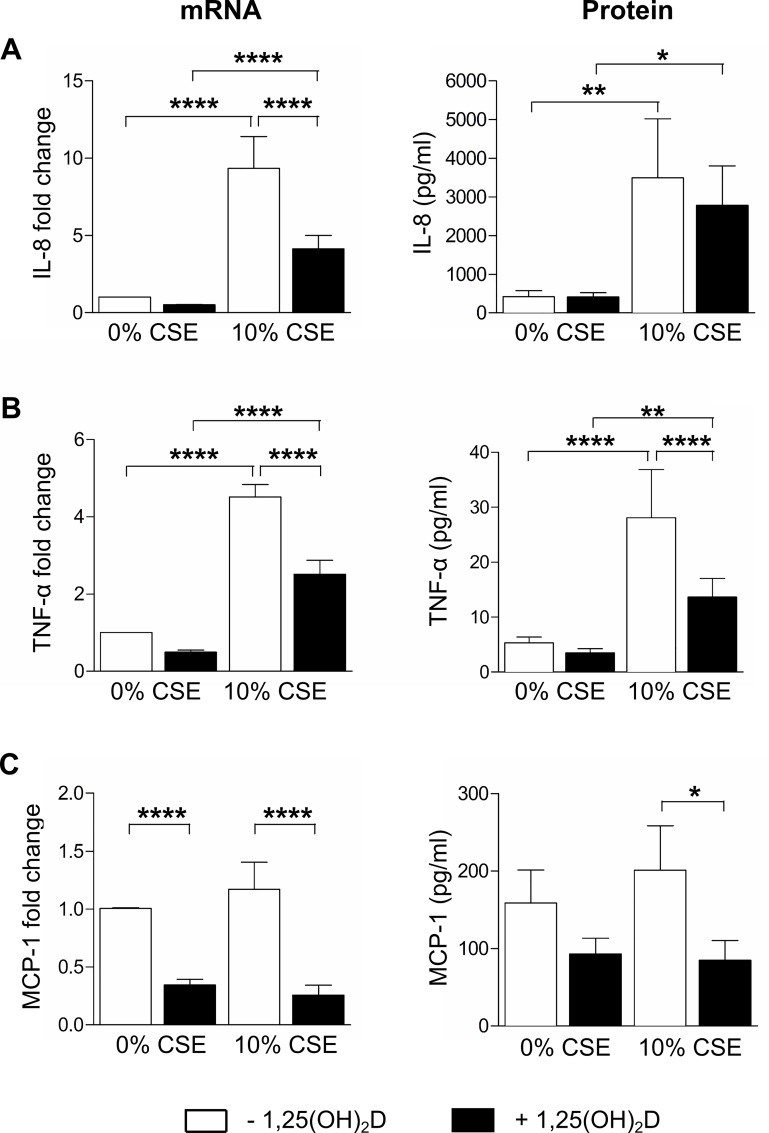
Effect of 1,25(OH)_2_D on the inflammatory response in CSE-treated THP-1 macrophages. THP-1 macrophages were stimulated for 16h with 10% CSE or vehicle, followed by an additional stimulation for 24h with 10 nM 1,25(OH)_2_D or vehicle. mRNA and protein levels of (A) IL-8, (B) TNF-α and (C) MCP-1 were determined in cell lysates and culture supernatants, respectively. Independent experiments were performed in triplicate. mean±SEM. *p<0.05, **p<0.01, ****p<0.0001.

**Fig 4:**
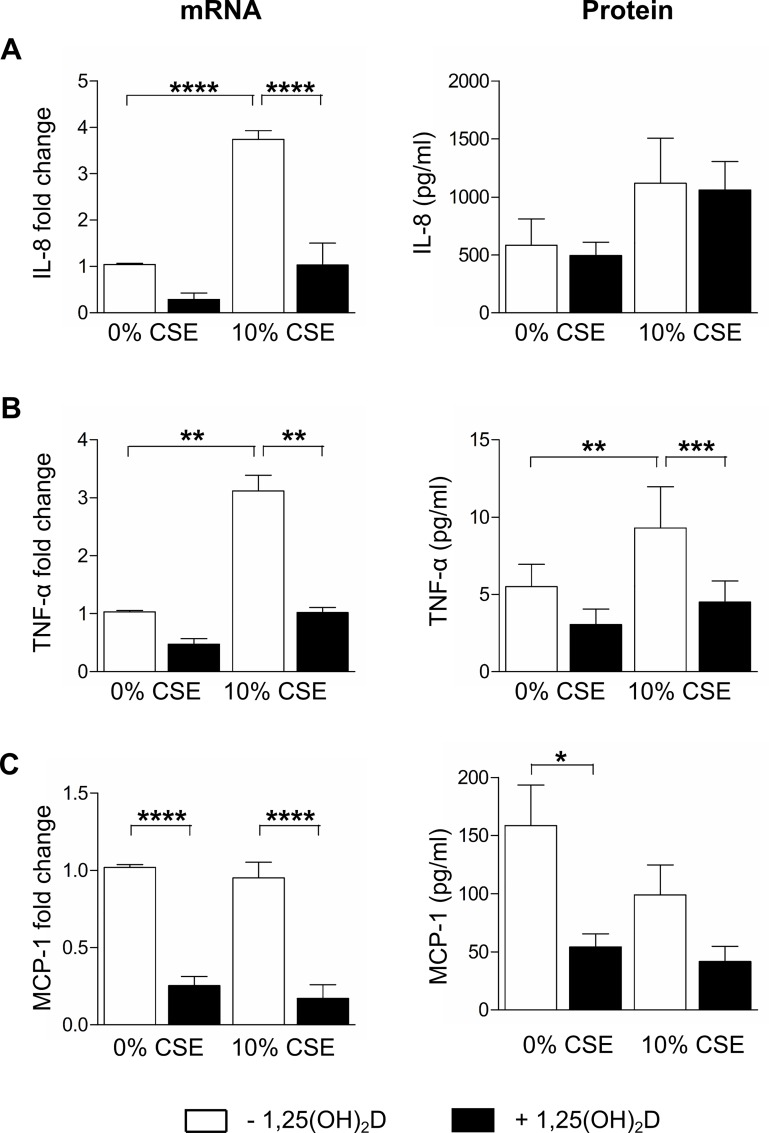
Effect of pre-treatment with 1,25(OH)_2_D on the inflammatory response of CSE-treated THP-1 macrophages. THP-1 macrophages were stimulated for 24h with 10 nM 1,25(OH)_2_D or vehicle, followed by an additional 16h stimulation with 10% CSE or vehicle. mRNA and protein levels of (A) IL-8, (B) TNF-α and (C) MCP-1 were determined in cell lysates and culture supernatants, respectively. Independent experiments were performed in triplicate. mean±SEM. *p<0.05, **p<0.01, ***p<0.001, ****p<0.0001.

As the number of BAL samples obtained from non-smoking or smoking subjects and the number of cells recovered from these samples was limited, a THP-1 macrophage cell line model for alveolar macrophages was used. To investigate whether 1,25(OH)_2_D may affect inflammatory responses in a context of CS exposure, THP-1 macrophages were exposed to 10 or 25% CSE as these CSE concentrations did not induce cytotoxicity (Figure A in S1 File). The effect of 1,25(OH)_2_D treatment on mRNA expression and protein levels of inflammatory cytokines and chemokines was assessed. Post-treatment with 1,25(OH)_2_D significantly reduced mRNA and/or protein levels of IL-8, TNF-α and MCP-1 compared to treatment with 10% CSE alone (Fig 3). Treatment with 1,25(OH)_2_D did not significantly alter protein levels of IL-8 induced by CSE (Fig 3). mRNA and protein levels of IL-6 could not be detected in all conditions. Experiments with 25% CSE showed similar results (data not shown). When pretreating THP-1 cells with 1,25(OH)_2_D prior to 10% CSE exposure, a similar reduction of inflammatory cytokines was also obtained (Fig 4).

### 1,25(OH)_2_D does not improve phagocytic or oxidative burst defects of THP-1 macrophages induced by CSE, but upregulates mRNA and protein levels of cathelicidin

**Fig 5:**
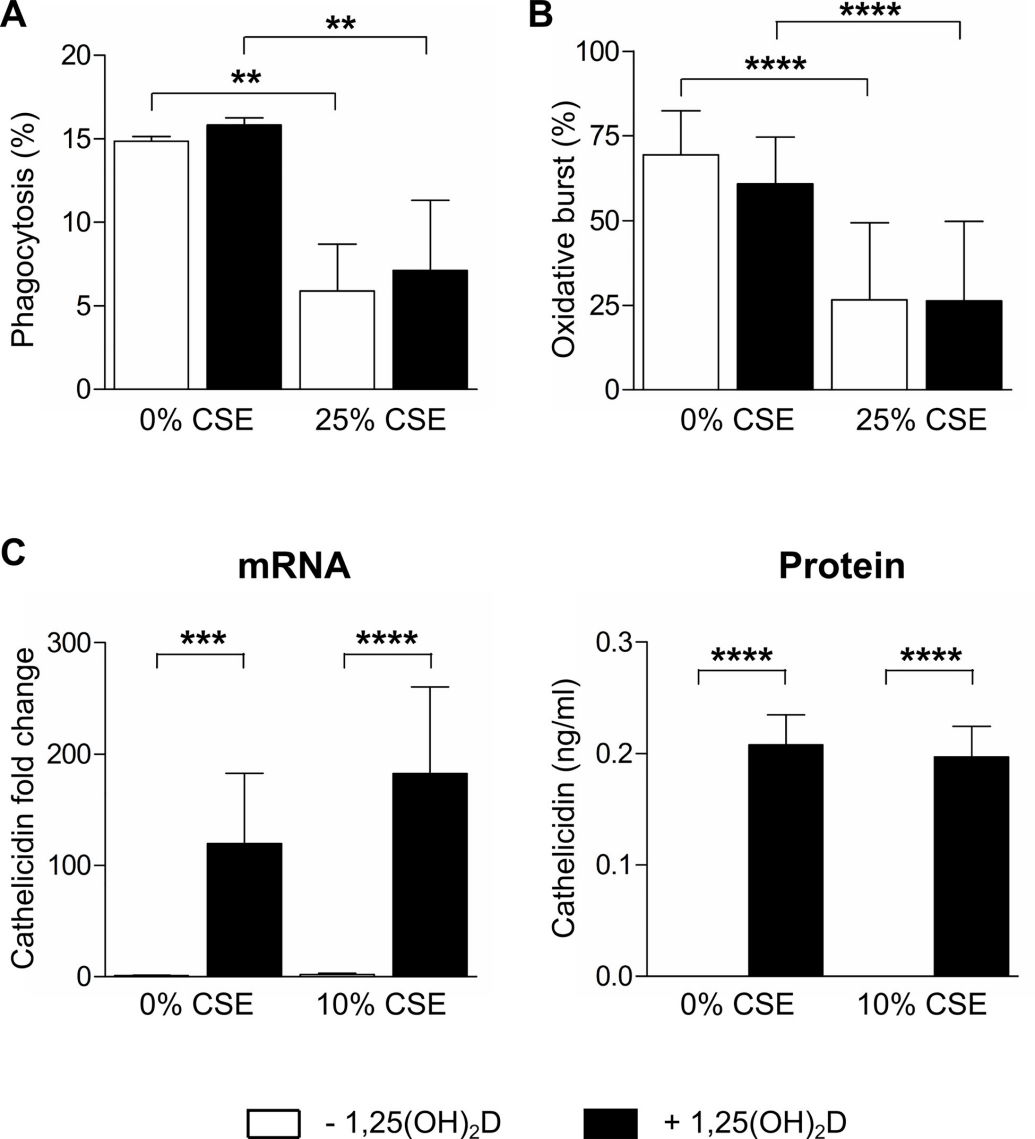
Effect of 1,25(OH)_2_D on antibacterial response in CSE-treated THP-1 macrophages. THP-1 macrophages were stimulated for 16h with 10% or 25% CSE or vehicle, followed by an additional stimulation for 24h with 10 nM 1,25(OH)_2_D or vehicle. (A) Phagocytosis and (B) oxidative burst by THP-1 macrophages following bacterial challenge. Graphs show the percentage of alveolar macrophages that have internalized *E*. *coli* bacteria (A) or have produced reactive oxygen species (B). (C) mRNA and protein levels of cathelicidin were determined in cell lysates and culture supernatants, respectively. Independent experiments were performed in triplicate. mean±SEM. **p<0.01, ***p<0.001, ****p<0.0001.

The phagocytic uptake of *E*. *coli* bacteria or oxidative burst by THP-1 macrophages was significantly decreased after exposure to 25% CSE (Fig 5A and 5B), but not 10% CSE (data not shown). However, 1,25(OH)_2_D did not improve phagocytosis or oxidative burst by THP-1 macrophages exposed to 25% CSE or vehicle (Fig 5A and 5B). On the other hand, 1,25(OH)_2_D significantly increased mRNA and protein levels of cathelicidin in THP-1 macrophages, which was not affected by the exposure to 10% CSE (Fig 4C) or 25% CSE (data not shown). All experiments on THP-1 macrophages were performed with 10% and 25% of CSE. As the results on cytokine release and concomitant suppression by 1,25(OH)_2_D were not different for both concentrations, we reported only the results obtained with 10% of CSE. However, to obtain a significant reduction of phagocytosis capacity 25% CSE was needed, without being toxic to these cells (Figure A in S1 File).

### Exposure to CSE does not alter expression levels of the activating or catabolizing enzyme of 1,25(OH)_2_D in THP-1 macrophages

**Fig 6:**
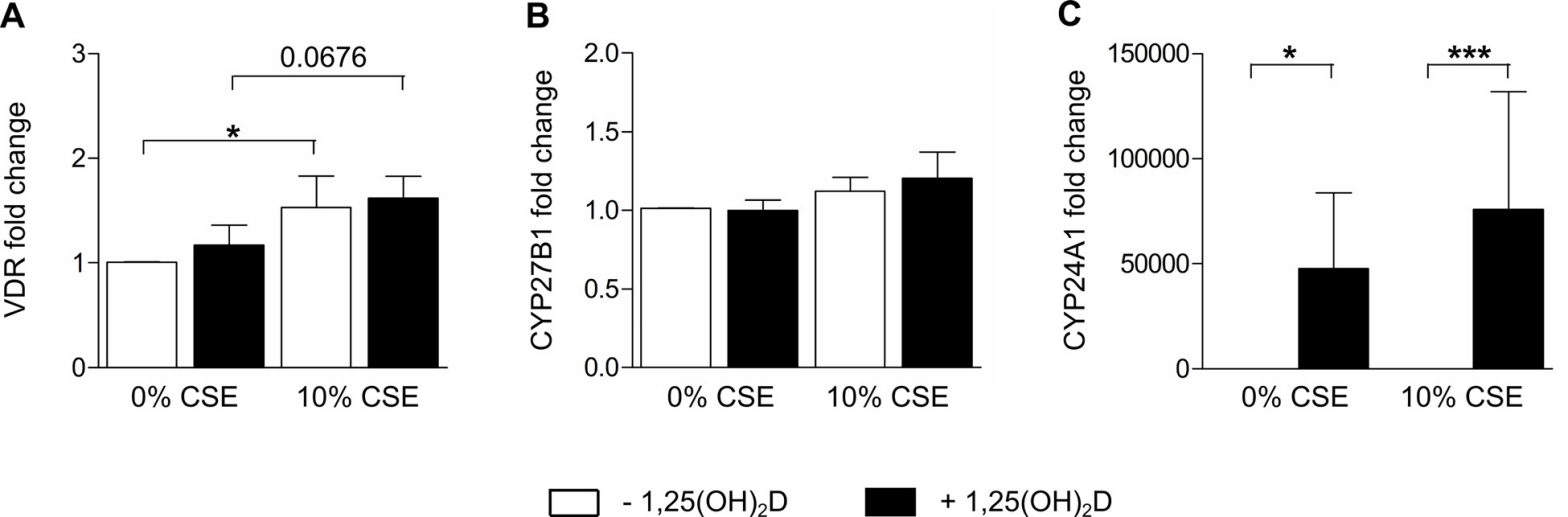
Effect of CSE on vitamin D metabolism in THP-1 macrophages. THP-1 macrophages were stimulated for 16h with 10% CSE or vehicle, followed by an additional stimulation for 24h with 10 nM 1,25(OH)_2_D or vehicle. mRNA expression levels of (A) VDR, (B) CYP27B1 and (C) CYP24A1 were determined in cell lysates. mean±SEM. *p<0.05, ***p<0.001

Finally, we wanted to investigate whether CSE may influence the expression of important proteins in the vitamin D pathway (CYP24A1, CYP27B1 or VDR) and in this way could limit anti-inflammatory or antibacterial actions of 1,25(OH)_2_D within CS-exposed airways. Stimulation with 1,25(OH)_2_D did not affect mRNA expression levels of VDR or CYP27B1, but significantly upregulated expression levels of CYP24A1, a known target for 1,25(OH)_2_D, in THP-1 macrophages (Fig 6). Treatment with 10% CSE did not significantly alter expression levels of CYP27B1 or CYP24A1 in THP-1 macrophages, although VDR expression levels were slightly, but significantly increased following treatment with 10% CSE (Fig 6). Experiments with 25% CSE showed similar results (data not shown).

## Discussion

*In vitro* research has demonstrated important antibacterial functions as well as anti-inflammatory effects of 1,25(OH)_2_D in response to different inflammatory stimuli. However, currently, it is not known whether vitamin D could influence inflammation and antibacterial defects in CS-exposed airways. As alveolar macrophages play a major role in innate immune processes within the respiratory system [12,13], we investigated in the present study whether 1,25(OH)_2_D may affect *ex vivo* responses of alveolar macrophages from smoking individuals. Unexpectedly, no differences were observed in baseline release of inflammatory mediators between alveolar macrophages from smokers and non-smokers, which may correlate with the low-grade baseline inflammation observed in the enrolled subjects, in whom COPD and other inflammatory pulmonary disease was excluded. To compare effects of 1,25(OH)_2_D on inflammatory responses between smoking and non-smoking subjects, alveolar macrophages were additionally stimulated with a strong inflammatory stimulus, a combination of LPS and IFN-γ, [25]. Interestingly, 1,25(OH)_2_D inhibited the release of pro-inflammatory cytokines (TNF-α, MCP-1 and IL-6) by alveolar macrophages from both non-smokers and smokers in response to LPS/IFN-γ stimulation. These results extend the idea that the anti-inflammatory potential of vitamin D in healthy individuals may also persist in smokers. However, due to small sample size, caution should be taken with general conclusions regarding our *ex vivo* data. In particular, the use of different concentrations of 1,25(OH)_2_D exposure but also different exposure times may possibly lead to slightly different results. To overcome the problem of a limited number of available macrophages per sample, we used PMA-stimulated THP-1 cells, which represent a useful model to study alveolar macrophage responses and which were previously shown to maintain a normal vitamin D metabolism [26,27]. In THP-1 macrophages, treatment with 1,25(OH)_2_D significantly reduced mRNA and/or protein levels of IL-8, TNF-α and MCP-1 in response to CSE. These results are in line with previous reports demonstrating the downregulation of pro-inflammatory cytokine production by 1,25(OH)_2_D in innate immune cells in response to inflammatory signals other than CS. In monocytes/macrophages, 1,25(OH)_2_D reduced the production of TNF-α and/or IL-6 in response to LPS by inhibiting NF-κB and p38 MAPK activity [2,5]. Moreover, Hansdottir and colleagues demonstrated that vitamin D attenuated the induction of IL-8 mRNA by viral mimetic poly(I:C) in airway epithelial cells [4]. Similarly, Golden and colleagues found that 1,25(OH)_2_D reduced IL-8 release by human bronchial epithelial cells and human peripheral blood monocytes induced by organic dust [3]. By lowering levels of cytokines and chemokines in response to CS, 1,25(OH)_2_D may indirectly reduce infiltration of inflammatory cells, including macrophages and neutrophils, into the airways and in this way downregulate the airway inflammatory response. Interestingly, in an animal model of acute lung injury, it has already been shown that intratracheal or peroral administration of 1,25(OH)_2_D inhibits the recruitment of neutrophils after LPS inhalation [28].

CS has been shown to impair antibacterial defense. *In vitro*, exposure to CSE reduced the phagocytic capacity of both macrophages and neutrophils [14,19]. Furthermore, we have previously shown that CS exposure of mice results in reduced *ex vivo* phagocytic and oxidative burst responses of alveolar macrophages in the BAL fluid [29]. In this study, we confirm that CS impairs antibacterial defense, as the phagocytic uptake of *E*. *coli* bacteria was significantly decreased in alveolar macrophages obtained from smoking subjects compared to non-smoking subjects. Similar trends were observed for the oxidative burst capacity of alveolar macrophages, although not statistically significant. Consistent with these results, both the phagocytic and oxidative burst capacity of THP-1 macrophages was reduced by exposure to CSE. These defects in the antibacterial functionality of macrophages may explain why cigarette smokers are more susceptible to respiratory infections [30]. However, in both human alveolar macrophages or THP-1 macrophages, 1,25(OH)_2_D did not significantly alter the phagocytic or oxidative burst capacity. In contrast to these findings, previous *in vitro* studies have demonstrated that 1,25(OH)_2_D can improve the phagocytic capacity as well as the oxidative burst functions of monocytes [6–9]. These effects of 1,25(OH)_2_D on monocyte antibacterial functions are potentially explained by the prodifferentiating effects of 1,25(OH)_2_D on monocytes, which adopt a more mature phenotype [6,22]. In addition, some of these studies have been performed with different cells (including P388D1 and phagocytic cells obtained from normal human peripheral blood). Although the dose used in our study (10^-8^M) was in the range that has been shown to have an effect on phagocytic capacity and oxidative burst, the different outcome may be explained by the use of different cell types. The effect of CSE on phagocytosis and oxidative burst was only clear when we used a CSE of 25% instead of the 10% reported for all other experiments with THP-1 cells. This generates the interesting hypothesis that the decrease in phagocytosis and oxidative burst only present at high levels of CSE could correlate with increased bronchial infections in heavy vs light smokers. However, to our current knowledge there are no reports in human of impaired macrophage function caused by cigarette smoke and its subsequent risk for infection. Although 1,25(OH)_2_D did not affect phagocytic or oxidative burst antibacterial functions, 1,25(OH)_2_D treatment significantly increased levels of the antimicrobial peptide cathelicidin released by alveolar macrophages from both smoking and non-smoking individuals. Consistently, we demonstrated that 1,25(OH)_2_D upregulates mRNA and protein levels of cathelicidin in THP-1 macrophages, independent of CSE exposure. Upregulation of cathelicidin by 1,25(OH)_2_D has been demonstrated in several cell types, including macrophages and neutrophils, and has shown to be crucial in the intracellular killing of *Mycobacterium tuberculosis* [10,11]. Moreover, cathelicidin exerts bactericidal activity to a broad spectrum of bacteria, including both Gram-positive and Gram-negative bacteria by disrupting bacterial membranes. Therefore, our results from both alveolar macrophages and THP-1 macrophages suggest that 1,25(OH)_2_D could improve antibacterial defense, also in a CS environment by upregulating cathelicidin, however, without affecting bacterial uptake or oxygen-dependent intracellular killing.

There are a few limitations of our study that need to be mentioned. Firstly, antibacterial defense was only assessed by measuring phagocytosis and oxidative burst following challenge with *E*. *coli* bacteria and mRNA expression and/or protein levels of cathelicidin. Although our data provide the first evidence of the antibacterial potential of 1,25(OH)_2_D in CS-exposed macrophages, it still needs to be explored whether the upregulation of cathelicidin also results in the effective clearance of more relevant bacterial species causing respiratory tract infections, such as nontypeable *Haemophilus influenzae* and *Streptococcus pneumoniae*, in cultures of CS-exposed cells using bactericidal assays. Secondly, the number of BAL samples and the number of cells recovered from these samples was limited. Therefore, not all outcomes could be assessed in cultures of human alveolar macrophages (e.g. mRNA expression levels of inflammatory mediators or proteins in the vitamin D pathway). Moreover, because of large variation between different human BAL samples, statistical significance was not always reached. Finally, we used CSE of which the physiological relevance in terms of tobacco smoke exposure is hard to interpret. Although we standardized the extracts to 10 or 25% concentrations based upon optical density, it is also hard to compare them with concentrations used in other studies.

A few studies have suggested that CS exposure may interfere with vitamin D metabolism and in this way with immunomodulatory functions of vitamin D within the respiratory tract. In this context, Mulligan *et al*. demonstrated that CSE downregulated CYP27B1 expression in control human sinonasal epithelial cells, leading to less conversion of 25OHD to 1,25(OH)_2_D [21]. Moreover, benzopyrene, a constituent of CS, was shown to enhance the 1,25(OH)_2_D-dependent induction of CYP24A1 in THP-1 cells [20]. In contrast, we did not find significant effects of CSE on expression levels of important proteins in the vitamin D pathway (VDR, CYP27B1 and CYP24A1) in THP-1 macrophages. In addition, preliminary data show no significant difference in CYP27B1 or CYP24A1 expression between the alveolar macrophages of non-smokers and smokers (data not shown). Although these findings should be confirmed in primary cells like respiratory epithelial cells from smoking subjects, our results suggest that vitamin D may provide a new strategy for attenuating inflammation and improving antibacterial defense in CS-exposed airways. Although our study did not specifically address the inflammation related to COPD, it is tempting to discuss our findings in the context of COPD, where CS exposure and vitamin D deficiency are strongly associated [22,31]. Moreover, different studies have demonstrated an accelerated decline of lung function (forced expiratory volume in 1 second, FEV1) and increased likelihood for COPD onset in smokers with vitamin D deficiency [32,33]. At this stage, there are no data showing that optimization of vitamin D status may prevent COPD onset, but two randomized placebo-controlled intervention trials have shown benefit of vitamin D supplementation in COPD patients with vitamin D deficiency with regard to exacerbations [34,35]. Therefore, our *in vitro* research may indirectly suggest that vitamin D supplementation, through the attenuation of macrophage-driven inflammation and the upregulation of cathelicidin levels, may reduce the risk of respiratory infections and COPD in smokers.

## Supporting Information

S1 File Supplementary methods and results. (DOCX)
