# Supplementary material for: 1,25-Dihydroxyvitamin D Modulates Antibacterial and Inflammatory Response in Human Cigarette Smoke-Exposed Macrophages
Source: PLoS One. 2016 Aug 11;11(8):e0160482. doi: 10.1371/journal.pone.0160482 (PMC4981391; doi:10.1371/journal.pone.0160482)
Supplement: S1 File — (DOCX) [file pone.0160482.s001.docx]

**S1 File**

**Methods**

**Stimulation of THP-1 macrophages**

PMA-stimulated THP-1 macrophages were stimulated as follows: i) for 16h with CSE, followed by an additional 24h stimulation with 1,25(OH)_2_D (1,25(OH)_2_D post-treatment) or ii) for 24h with 1,25(OH)_2_D, followed by an additional 16h stimulation with CSE (1,25(OH)_2_D pre-treatment).

**Results**

**Effect of CSE on cytotoxicity in THP-1 macrophages**

THP-1 macrophages were either stimulated for 16h (1,25(OH)_2_D pre-treatment) or 40h (1,25(OH)_2_D post-treatment) with CSE. To determine working CSE concentrations, THP-1 macrophages were incubated for 16h or 40h with different concentrations of CSE, ranging from 0 to 100%, and cytotoxicity was evaluated. Cell viability was not affected by CSE concentrations up to 50% following 16h or 40h incubation (S1 Fig). Future experiments were performed with CSE concentrations of 10 or 25%, which were confirmed not to induce cytotoxicity.


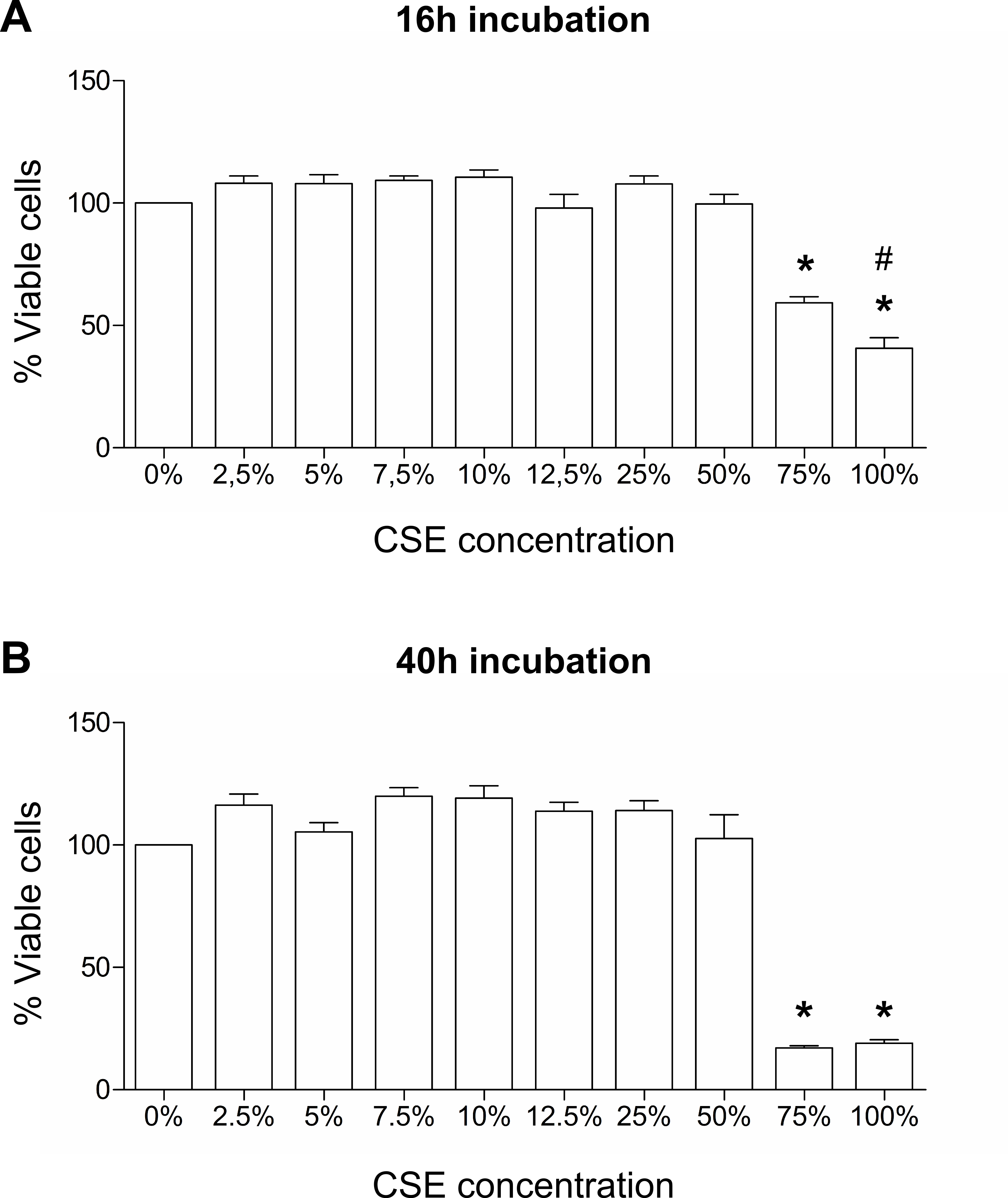


**S1 Fig. Effect of CSE on cytotoxicity in THP-1 macrophages.** THP-1 macrophages were incubated for (A) 16h or (B) 40h with different concentrations of CSE and cytotoxicity was evaluated using the MTT assay. Results are expressed as percentage of viable cells compared to control values. Independent experiments were performed in triplicate. mean±SEM. *p<0.0001 vs CSE 0-50%, ^#^p<0.05 vs CSE 75%.
